# Dynamics of malaria vector composition and *Plasmodium falciparum* infection in mainland Tanzania: 2017–2021 data from the national malaria vector entomological surveillance

**DOI:** 10.1186/s12936-024-04849-7

**Published:** 2024-01-19

**Authors:** Charles D. Mwalimu, Samson Kiware, Rosemary Nshama, Yahya Derua, Pendael Machafuko, Peter Gitanya, Winfred Mwafongo, Jubilate Bernard, Basiliana Emidi, Victor Mwingira, Robert Malima, Victoria Githu, Brian Masanja, Yeromin Mlacha, Patrick Tungu, Bilali Kabula, Edward Sambu, Bernard Batengana, Johnson Matowo, Nicodem Govella, Prosper Chaki, Samwel Lazaro, Naomi Serbantez, Jovin Kitau, Stephen M. Magesa, William N. Kisinza

**Affiliations:** 1 National Malaria Control Programme (NMCP), Dodoma, United Republic of Tanzania; 2 Ifakara Health Institute (IHI), Dar es Salaam, Tanzania; 3 Pan African Mosquito Control Association (PAMCA), Dar es Salaam, Tanzania; 4 National Institute for Medical Research (NIMR), Amani Centre, Muheza, Tanzania; 5 National Institute for Medical Research (NIMR), Mwanza, Tanzania; 6 University of Dar es Salaam, Mbeya College of Health and Allied Sciences, Mbeya, Tanzania; 7 Department of Medical Parasitology and Entomology, Kilimanjaro Christian Medical University College, Moshi, Tanzania; 8 Population Services International (PSI), Dar es Salaam, Tanzania; 9 U.S. President’s Malaria Initiative, Dar es Salaam, Tanzania; 10 World Health Organization, Country Office, Dar es Salaam, Tanzania

**Keywords:** Surveillance, Malaria vectors, Entomological inoculation rate (EIR), Integrated vector management, Tanzania

## Abstract

**Background:**

In 2015, Tanzania National Malaria Control Programme (NMCP) established a longitudinal malaria vector entomological surveillance (MVES). The MVES is aimed at a periodical assessment of malaria vector composition and abundance, feeding and resting behaviours, and *Plasmodium falciparum* infection in different malaria epidemiological strata to guide the NMCP on the deployment of appropriate malaria vector interventions. This work details the dynamics of malaria vector composition and transmission in different malaria epidemiological strata.

**Methods:**

The MVES was conducted from 32 sentinel district councils across the country. Mosquitoes were collected by the trained community members and supervised by the NMCP and research institutions. Three consecutive night catches (indoor collection with CDC light trap and indoor/outdoor collection using bucket traps) were conducted monthly in three different households selected randomly from two to three wards within each district council. Collected mosquitoes were sorted and morphologically identified in the field. Thereafter, the samples were sent to the laboratory for molecular characterization using qPCR for species identification and detection of *P. falciparum* infections (sporozoites). ELISA technique was deployed for blood meal analysis from samples of blood-fed mosquitoes to determine the blood meal indices (BMI).

**Results:**

A total of 63,226 mosquitoes were collected in 32 district councils from January 2017 to December 2021. Out of which, 39,279 (62%), 20,983 (33%) and 2964 (5%) were morphologically identified as *Anopheles gambiae *sensu lato (s.l.), *Anopheles funestus* s.l., and as other *Anopheles* species, respectively. Out of 28,795 laboratory amplified mosquitoes, 13,645 (47%) were confirmed to be *Anopheles arabiensis,* 9904 (34%) as *An. funestus *sensu stricto (s.s.), and 5193 (19%) as *An. gambiae* s.s. The combined average entomological inoculation rates (EIR) were 0.46 (95% CI 0.028–0.928) for *An. gambiae* s.s., 0.836 (95% CI 0.138–1.559) for *An. arabiensis*, and 0.58 (95% CI 0.165–0.971) for *An. funestus* s.s. with variations across different malaria transmission strata. *Anopheles funestus* s.s. and *An. arabiensis* were predominant in the Lake and South-Eastern zones, respectively, mostly in high malaria transmission areas. Monthly mosquito densities displayed seasonal patterns, with two peaks following the rainy seasons, varying slightly across species and district councils.

**Conclusion:**

*Anopheles arabiensis* remains the predominant vector species followed by *An. funestus* s.s. in the country. Therefore, strengthening integrated vector management including larval source management is recommended to address outdoor transmission by *An. arabiensis* to interrupt transmission particularly where EIR is greater than the required elimination threshold of less than one (< 1) to substantially reduce the prevalence of malaria infection.

## Background

Malaria is still a major cause of illness in about 85 countries worldwide, with an estimated 249 million cases in 2022 [1]. Globally, malaria cases have increased by 7.39% from the baseline year of the Global Technical Strategy for malaria (2016–2030) [1, 2]. However, malaria incidence per 1000 population at risk has decreased from 82 in 2000 to 59 in 2020, during which four African countries contributed to almost half of the global malaria cases [1]. Likewise, malaria mortality per 100,000 population at risk halved (decreased by 50%) between 2000 and 2021; nevertheless, four African countries, including Tanzania, accounted for over half of all malaria deaths globally in 2021 [1]. Despite important and diverse efforts towards control, malaria remains a challenge to public health particularly in sub-Saharan Africa (SSA) [1, 3].

In Africa, two groups of mosquitoes of the genus *Anopheles* transmit human malaria parasites [4], namely, the *Anopheles gambiae* complex and the *Anopheles funestus* group [4, 5]. Among the *An. gambiae* complex, *An. gambiae *sensu stricto (s.s.) and *Anopheles coluzzii* are the most efficient malaria vectors in SSA. *Anopheles funestus* s.s. is typically the most anthropophilic and endophilic member of the group and is a highly efficient vector of malaria [4]. This species is widespread throughout subtropical Africa, extending from northern Sudan to South Africa including Tanzania [4, 6]. Long-lasting insecticidal nets (LLINs) and indoor residual spraying (IRS) are the primary insecticide-based vector control interventions targeting predominantly indoor biting malaria vectors [4, 7, 8]. In Tanzania, LLINs are the most widely used malaria vector control intervention and have contributed to the decline in malaria transmission and burden in the period between 2005 and 2015, especially in settings with moderate to high malaria transmission [1]. The implementation of insecticide-based malaria vector control interventions has led to the rapid emergence of both physiological and behavioural resistance mechanisms in many vector populations in Africa [7].The spread of resistance mechanisms and changes in the vector population composition poses a major challenge to malaria vector control and thus threatens malaria control efforts in SSA [9–11]. Resistance to insecticides used in vector control interventions, such as LLINs and IRS, reduces their effectiveness in targeting and eliminating mosquito populations. Additionally, different species of mosquitoes may exhibit variations in their biting preferences, resting habits, and susceptibility to insecticides [12]. These variations and any alterations as a result of the control interventions are likely to influence the success of control measures such as LLINs and IRS. Therefore, it is essential to monitor malaria vector bionomics, resistance, and contribution thereof to malaria transmission in areas where vector control measures are implemented [7, 13, 14]. Understanding the diverse behaviour and characteristics of mosquito vectors is essential to develop targeted interventions that can effectively interrupt their life cycle and reduce transmission. By conducting vector surveillance, predominant species in specific areas, their preferred host species, and their susceptibility to insecticides can be determined [7, 11]. A comprehensive report on trends of insecticide resistance in mainland Tanzania from 2004 to 2020 is provided by Tungu et al. [15].

In 2016, National Malaria Control Programme (NMCP) established longitudinal national Malaria Vector Entomological Surveillance (MVES) to monitor vector species composition, their abundance and seasonality, feeding and resting behaviour to guide deployment of appropriate vector control interventions and assess their performance overtime. The implementation of MVES was a collaborative effort between NMCP and President’s Office Regional Administration and Local Government (PO-RALG), National Institute for Medical Research (NIMR) and financially supported by the Global Fund (GF). The MVES aimed at periodically assessing malaria vector species composition, their abundance and seasonality, feeding and resting behaviour to guide deployment of appropriate vector control interventions and assess their performance overtime. The establishment of functional MVES was in line with the Global Technical Strategy for Malaria (2016–2030), which emphasizes on strengthened and sustained epidemiological and entomological surveillance systems through substantial long term financial and political commitment [2].

To drive further progress against malaria in the face of dwindling resources, Tanzania administrative district councils were recently classified epidemiologically (stratified) based on the malaria risk into very low, low, moderate, and high and one operational stratum-urban [16]. Malaria stratification is the country’s attempt to tailor intervention approaches to optimize impact and cost-effectiveness. The malaria transmission risk stratification has become a lens through which programmatic implementation, progress and impact is viewed and/or assessed.

The current study presents the findings of MVES based on the vector abundance, species compositions, behaviours, sporozoite rate, and entomological inoculation rate (EIR) of malaria vectors across different malaria transmission strata in mainland Tanzania [16]. The findings also highlight the challenges experienced, lessons learnt, and solutions implemented to strengthen MVES implementation.

## Methods

### Study area

The United Republic of Tanzania lies between 1 and 12 degrees south of the equator and 29–41 degrees east and has a tropical climate. According to the 2022 national population and housing census, the country has 61,741,120 people [17]. Malaria burden in Tanzania varies across geographical regions. Based on the malaria risk, administrative district councils were classified epidemiologically into four strata namely very low, low, moderate, and high with parasite prevalence of < 1, between 1 and 5, between 5 and 30 and ≥ 30 respectively[16].

Tanzania is characterized by diverse topographical features extending from the coastal belt of the Indian Ocean with an extensive plateau and elevation ranging from 1000 m to 2000 m above sea level. The country experiences unimodal and bimodal rainfall, depending on the elevation. The northern parts of the country, including areas around the Lake Victoria Basin, northern coast, and areas around Mount Kilimanjaro experience two rain seasons (bimodal rainfall); with a long rainy season from March to May and a relatively shorter one from October to December. In this region, the annual rainfall averages varies from 550 mm in the central part up to 3690 mm in some parts of south-western highlands [18]. On the other hand, the Central, Southern and Western parts of Tanzania are characterized by one rainy season (unimodal rainfall) that occurs between November and April. The temperature ranges between 10 and 20 degrees Celsius (°C) in the highlands and is usually higher than 20 °C in the lowlands throughout the year. The hottest months are November to February, while the coldest are May to August.

### Study design

**Fig. 1:**
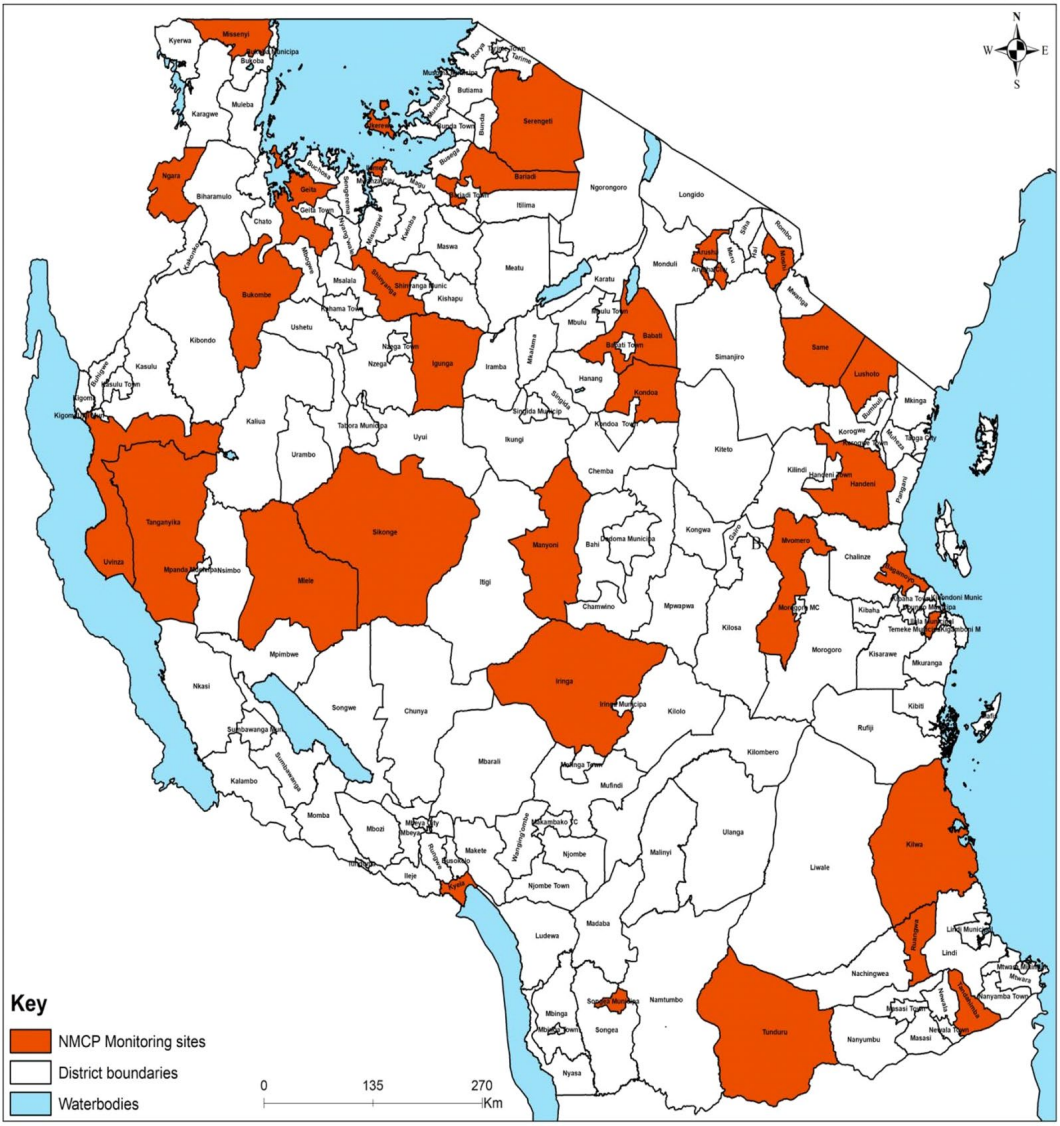
MVES programme implementation in the mainland: 32 district councils in 23 regions in Tanzania

The longitudinal MVES programme was initiated in 2016 with 62 district councils in 26 regions in mainland Tanzania. However, due to financial reasons, in 2021 the surveillance sites were reduced to 32 district councils in 23 regions (Fig. 1). MVES focuses on establishing the dynamics of malaria vector composition, sporozoite rates, and entomological inoculation rates across different malaria transmission strata. To date, MVES is conducted in 32 district councils comprising all four malaria transmission strata.

### Sampling framework

A multistage sampling technique was used to select representative district councils based on criteria outlined above from 23 regions of mainland Tanzania. Two district councils were selected from each region. The selection criteria included district councils implementing major malaria vector control interventions (i.e., LLINs, IRS), those bordering with other countries, malaria endemicity, land use pattern (e.g., irrigation), and demographic characteristics (e.g., rural vs urban).

**Fig. 2:**
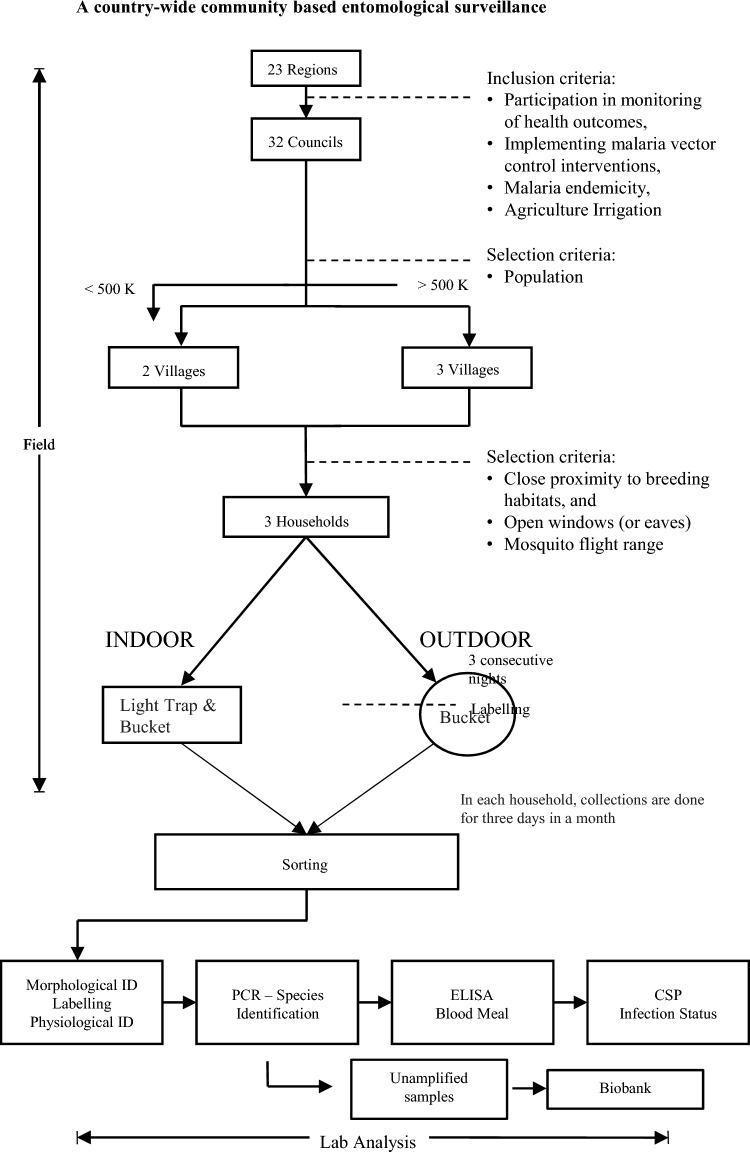
Schematic illustration on the country wide malaria vector entomological surveillance and laboratory analysis

The villages (sentinel sites) for adult mosquito surveillance were determined based on the population size and type of district council. For district councils with a population of at least 500,000, three villages were selected while those with a population of less than 500,000 two villages were selected (Fig. 2). A total of 65 villages were selected out of 32 districts. The district councils were divided into strata (administrative division) equal to the number of study sites required. Each district council was divided into three to six divisions according to the population and geographical areas. The distance between the selected sites within a district council was ≥ 30 km with exceptions for urban settings and districts with small areas.

### Household selection, adult mosquito collection and processing

In each village, three households were selected for mosquito sampling based on proximity to breeding habitats, and house characteristics including presence of open eaves/windows. Adult mosquitoes were sampled both indoors and outdoors simultaneously for three days consecutively per month. The indoor collection was done indoors with only the battery-powered Centers for Disease Control (CDC) light traps described in [19–22], but indoors and outdoors using bucket traps where resting mosquitoes were sampled [23]. The traps were set by community volunteers under the supervision of District Vector Control Officers (DVCOs). CDC light traps were hung at the foot-end of the bed at 1.5 m above the floor with an adult person sleeping under treated mosquito net from 18:00 h to 06:00 h. The trapped mosquitoes were retrieved in the morning from 6:00 h to 07:00 h. The setting and retrieval time of bucket traps were done at the same time as the CDC light traps. Mosquitoes from each collection method in the field were sorted, morphologically identified, and stored in paper cups with pre-identified date and location of collection by DMVCOs. Females of the *An. gambiae* complex and the *An. funestus* group were kept individually in 1.5 ml polypropylene Eppendorf tubes with silica gel desiccants. Male mosquitoes were discarded. Preserved mosquito samples were shipped to the National Institute for Medical Research Amani Research Centre for further laboratory analyses. A team consisting of Vector Control Technical Working Group members led by the NMCP conducted supervision of the field activities at least once every three months across all sentinel sites.

### Laboratory analyses

Prior to molecular analyses, mosquito samples were identified morphologically under stereo microscopy by NIMR Amani Research Centre technicians as part of quality assurance using standard keys [24] to verify the morphological identifications done in the field by the DVCOs. Wrongly packed, non-*Anopheles* mosquitoes and male mosquitoes were discarded. Female anopheline were sorted according to abdominal status as unfed, fed, half gravid and gravid. All identified female members of *An. gambiae* complex and *An. funestus* group were transferred to new 1.5 ml Eppendorf tubes for further analyses including molecular characterisation.

### Density of adult anopheline mosquitoes

The density of adult Anopheline mosquitoes was calculated as the number of female mosquitoes per trap/night for each collection method. The proportions of sibling species of Anopheline mosquitoes were calculated as the number of each species over the total Anophelines collected and results were presented in bar charts. The results were disaggregated by year and month of collection, collection methods, malaria epidemiological strata and sentinel district councils.

### Identification of sibling species

In the laboratory, malaria vectors were identified into respective sibling species by polymerase chain reaction (PCR). Genomic DNA (gDNA) was extracted from the leg of either *An. gambiae* complex or *An. funestus* group by hot sodium hydroxide and Tris (HotSHOT) method as described elsewhere [25]. Members of the *An*. *gambiae* complex were identified by PCR based on the method previously described to identify members of *An. gambiae* complex, namely *An*. *gambiae* s.s., *Anopheles arabiensis*, *Anopheles quadriannulatus*, *Anopheles melas, Anopheles bwambae* and *Anopheles merus* [26]. On the other hand, sibling species of the *An*. *funestus* group were identified based on species-specific primers targeting ribosomal DNA genes, a method previously described to identify *An*. *funestus* s.s., *Anopheles vaneedeni*, *Anopheles rivulorum*, *Anopheles leesoni* and *Anopheles parensis* [4].

### Detection of sporozoite infection in malaria vectors

DNA extracted from the head and thorax of the adult females *Anopheles* was analysed for *Plasmodium* sporozoite infection using PCR targeting *cytochrome oxidase I* (*cox-1*) gene, as previously described [27]. The sporozoite rate was estimated as the proportion of mosquitoes positive for *Plasmodium falciparum* by PCR out of the total number of mosquitoes tested.

### Mean biting rates, sporozoite rate and entomological inoculation rate

Sporozoite rate (SR) is the fraction of vector mosquitoes that are considered infectious, expressed as a percentage, while man biting rates (MBR) is the number of vectors biting an individual over a fixed period of time. The entomological inoculation rate (EIR) is the number of infectious bites per person per unit time, usually measured or expressed per year. Hence, EIR is the product of the human biting rate and the sporozoite rate. the annual EIR was estimated for malaria vectors sampled using CDC light traps by multiplying 1.605 × (number of sporozoite positive PCRs/number of mosquitoes tested) × (number of mosquitoes collected CDC light traps/trap nights) × 365 [26, 28, 29]. The multiplication factor 1.605 is a conversion factor for comparing estimate for CDC light trap with standard human landing catch [22]. The annual EIR was calculated separately for each malaria vector species and Tukey test was performed on one-way ANOVA to test for statistical difference between species and epidemiological strata. Confidence intervals on the EIR were computed using a bootstrap approach where samples were bootstrapped and the 2.5th and 97.5th quantiles used for the confidence limits. Only district councils with monthly consistency in data submission were included in the computing for the calculation of annual EIR. Thus, a total of 14 district councils were included in the computing the EIR.

### Data management, processing, cleaning, and analysis

The data processing, cleaning and analysis of malaria vector entomological surveillance included all mosquito data gathered between January 2017 to December 2021 in 32 district councils. MS Excel data collection form was used to gather data at the outset of the surveillance where DVCO recorded/filled forms were sent to the NMCP coordinator via email. Beginning in June 2021, a modified data collection form with re-organization of the columns, addition of new columns and locking of cells was used to improve data quality. However, this change of collection form did not distort the value of the data collected using the previous format, it was just an improvement of the data collection sheet. The data files from both templates were imported into the R programming statistical language and renamed with new variable names from the dictionary in preparation for cleaning and analysis.

### Field data cleaning

The field data was examined for typos, inaccurate and irrelevant values, missing observations, and duplicates. The mistakes included the names of regions, district councils, wards, and trapping techniques; the wrong and irrelevant elements were replaced with their respective accurate values from the source document. Prior to data analysis, the R scripts describing the cleaning process for the two Excel versions were constructed and the final clean datasets were added (combined) to a new complete dataset. Outlying data points were also removed through a visual inspection and confirmation for each district council. The field and laboratory data were then cross checked based on district councils with consistent monthly data submission in the laboratory to perform the required analysis for species composition, blood meal sources and sporozoite infection. The time series were provided based on field data where mosquitoes were identified morphologically to assess the distribution of *An. gambiae* complex and *An. funestus* group over time. Rainfall data was obtained from Tanzania meteorological agency (TMA) and was overlaid on mosquito species to show the association between rainfall patterns and mosquito data over time.

## Results

### Collection of *Anopheles* mosquitoes

A total of 63,226 *Anopheles* mosquitoes were collected from 32 sentinel district councils from 2017 to 2021. Morphological identification of the collected mosquitoes revealed that 39,279 (62.1%) were *An. gambiae* complex, 20,983 (33.2%) were *An. funestus* group and 2964 (4.7%) were non-malaria *Anopheles* species. Non-malaria vectors were recorded but not included for further analysis.

### Mosquito catches by traps

**Fig. 3:**
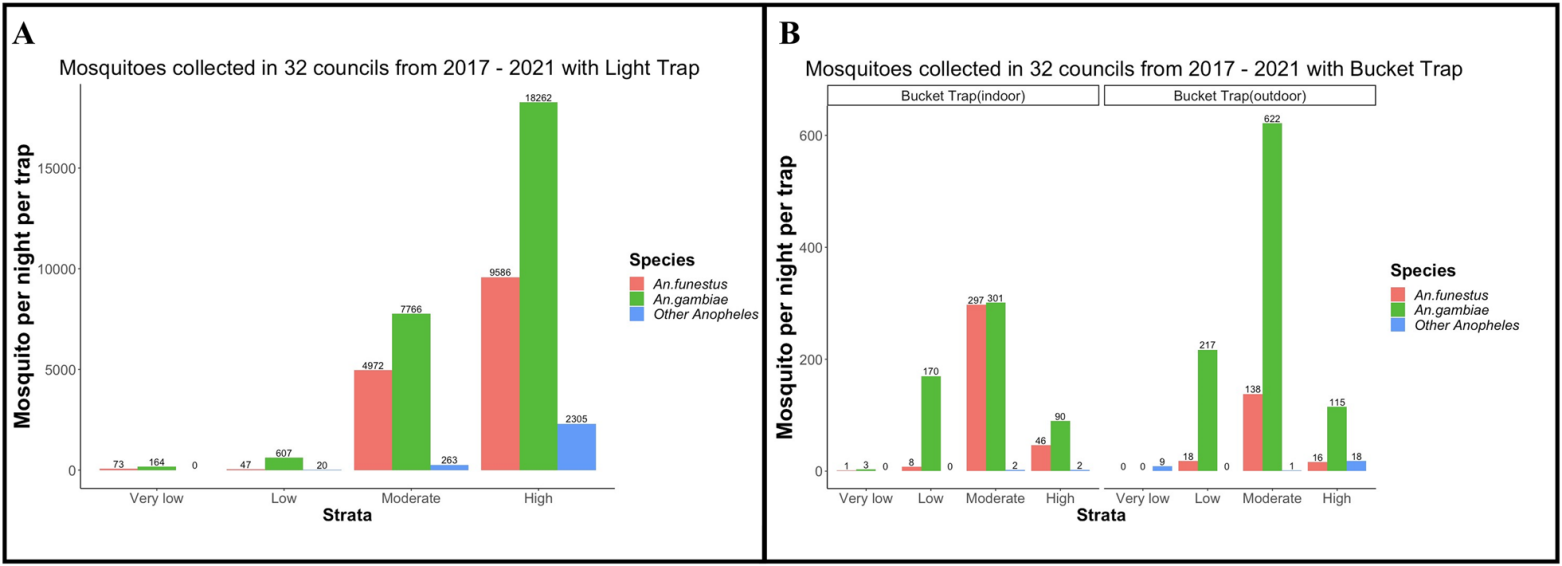
Mosquito catches per night per trap: **A** CDC light traps in different strata by species and **B** Bucket trap (indoor vs outdoor) in different strata by species

For a total of 180 trapping nights throughout the epidemiological strata, CDC light traps caught more mosquitoes than the bucket traps (Fig. 3A). Regardless of the low numbers collected in a bucket trap used as a proxy for mosquito abundance, *An. gambiae *sensu lato (s.l.) was more predominant outdoors than indoors while *An. funestus* s.l. was more predominant indoors than outdoors (Fig. 3B). Additionally, the moderate transmission strata had the highest mosquito density of the four transmission strata in the bucket trap collections (Fig. 3B).

### Malaria vector species composition and dynamics based on laboratory analysis from all 32 district councils

**Fig. 4:**
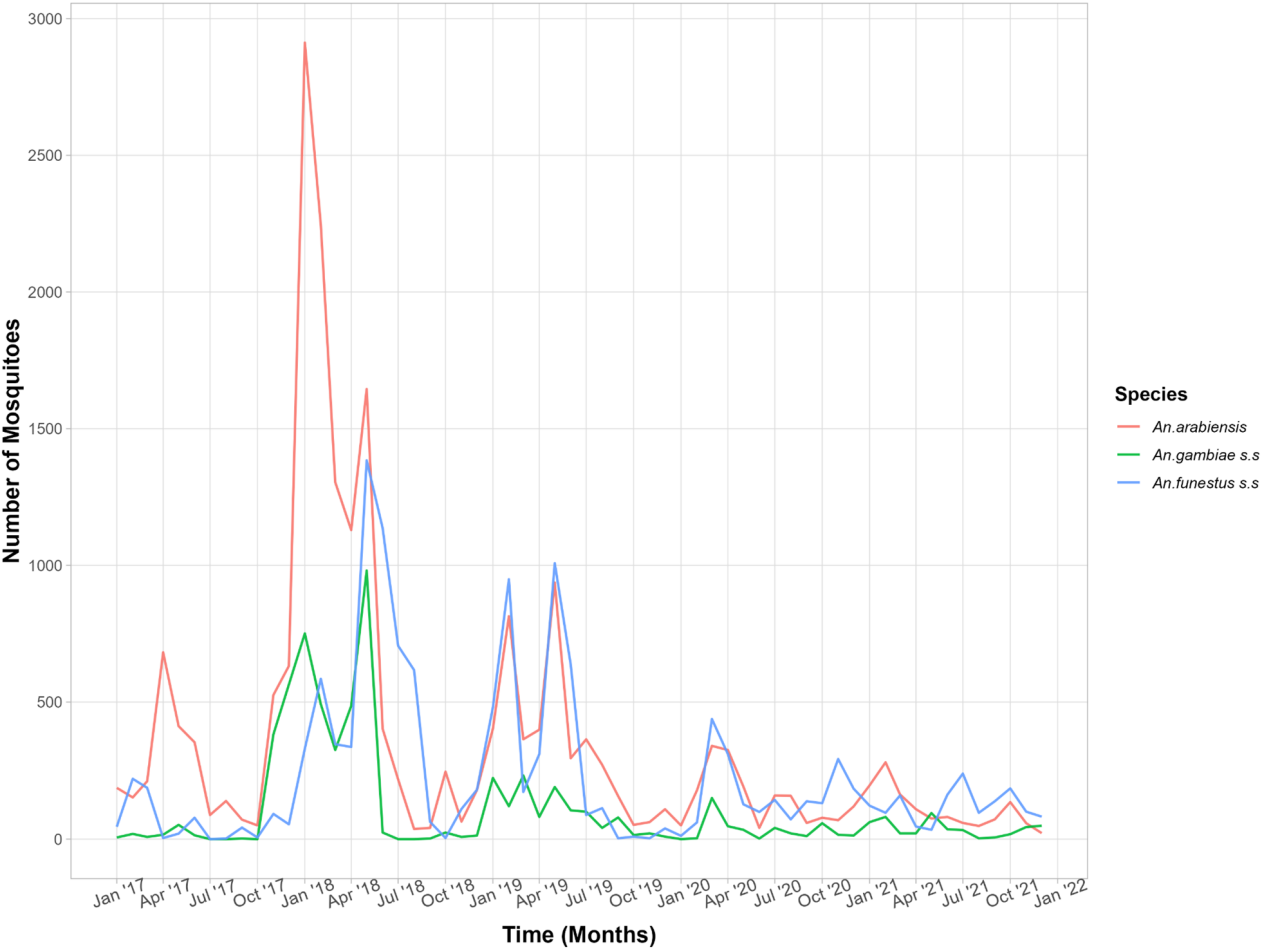
Malaria vector species composition over time from 2017 to 2021 combined data from 32 district councils in Tanzania

Malaria vector species composition was assessed in all district councils that submitted mosquito samples for laboratory analysis regardless of the consistency in monthly submission. However, feeding preference, sporozoite rates, and entomological inoculation rates are only reported for district councils that submitted samples consistently each month from 2017 to 2021. The trends over time were consistent for all *Anopheles* species when data from all district councils were combined (Fig. 4). In recent years, numbers of *An. arabiensis* were slightly higher than *An. funestus* s.s., unlike *An. gambiae* s.s. (Fig. 4). A general decline in mosquito density over time was also observed for all the three major species.

**Fig. 5:**
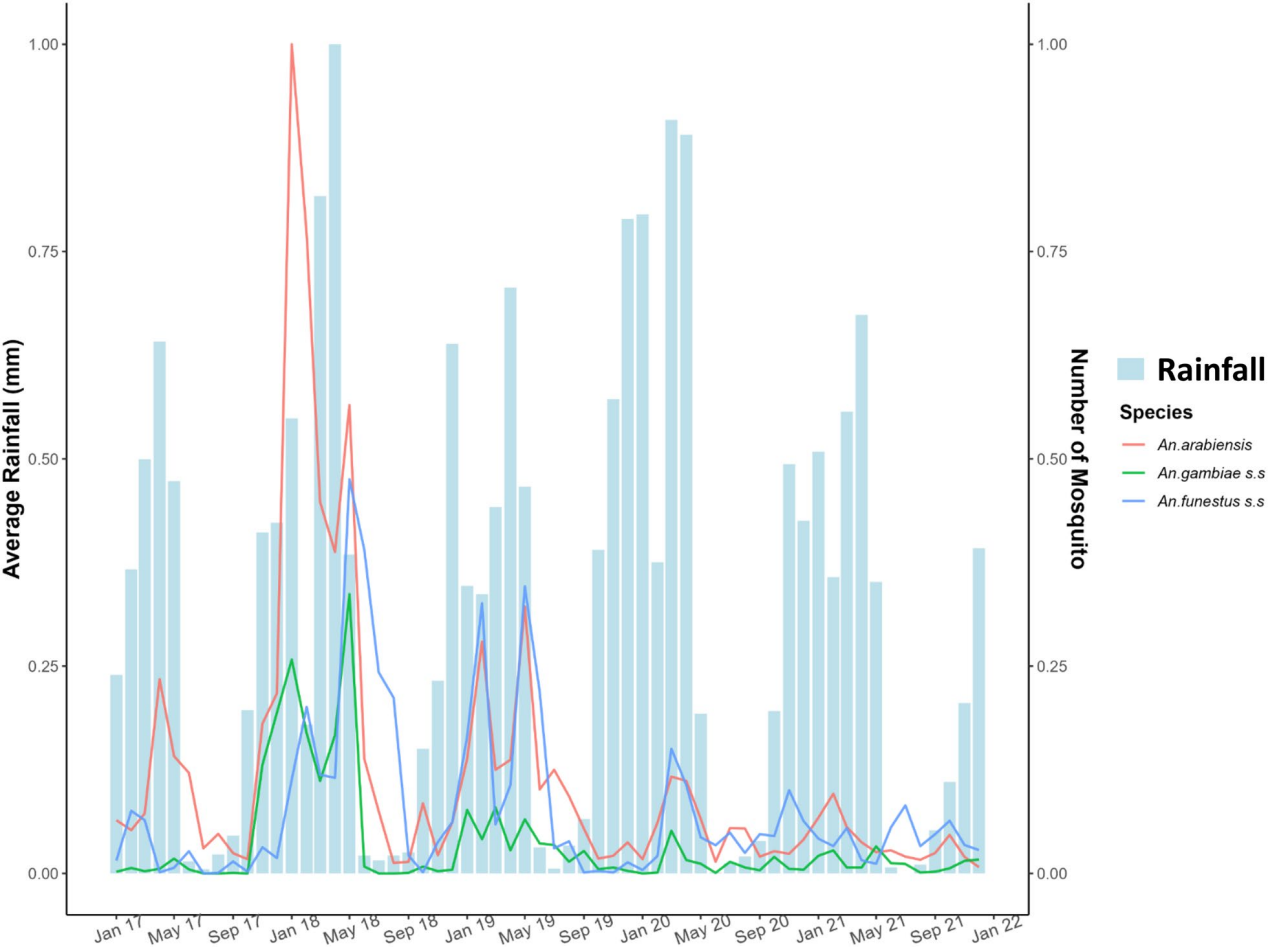
An illustration of mosquito densities varying according to rainfall patterns for the year 2017–2021

Tanzania experiences short (November to mid-January) and the long (mid-March to May) rainy seasons with considerable variations between district councils. Densities of *An. arabiensis* peaked in February (after the short rains) and May (after the long rains) while *An. funestus* s.s. peaked in May after the long rainy season (Fig. 5) and *An. gambiae* s.s. peaked in October and February. The lowest malaria vector densities were observed between June and August with minor variation between district councils. The rainy and mosquito data is normalized for Fig. 5 to show the variation between the two.

### Species composition per different transmission strata for all 32 District councils

**Fig. 6:**
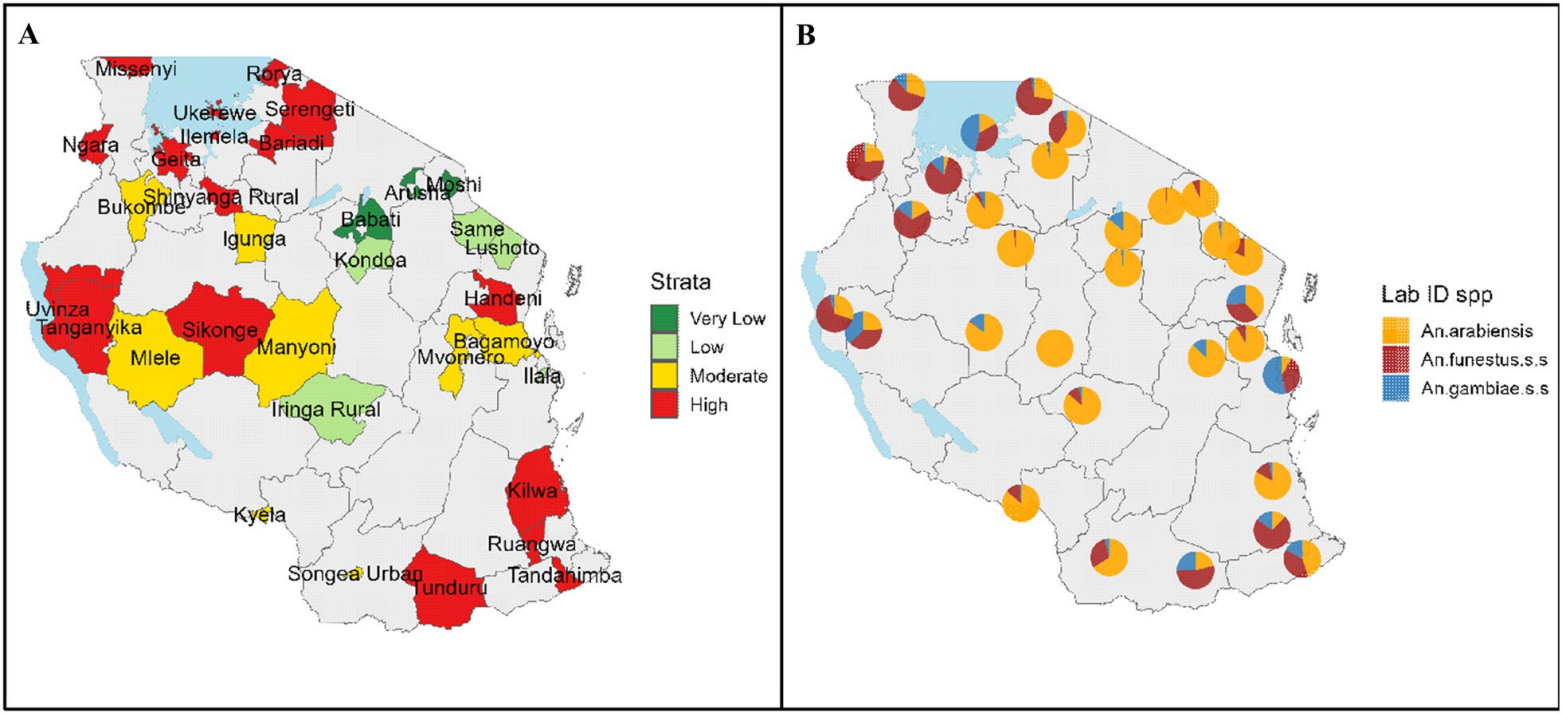
Malaria vector species composition across different transmission strata in 32 district councils: malaria transmission strata (**A**) for each district council with corresponding species composition (**B**)

Out of 41,383 mosquitoes that amplified during laboratory testing based on samples submitted from 32 district councils, 21,218 (51%) were *An. arabiensis,* 13,825 (33%) *An. funestus* s.s*.* and 6250 (15%) *An. gambiae* s.s*.* Other *Anopheles* species identified although in very low proportions include *An. merus,* other *An. funestus* species, *An. parensis, An. quadrianulatus*, and *An. leesoni* (Fig. 6).

### Malaria vector species composition and dynamics based on laboratory analysis out of 14 district councils

Based on 14 district councils that consistently submitted samples for analysis, 47% of the 28,795 samples that tested successfully, were *An. arabiensis*, 34% *An. funestus* s.s., and 18% were *An. gambiae* s.s. (Fig. 6). *Anopheles merus*, *An. parensis, An. quadrianulatus*, and *An. leesoni* were also identified, but at proportions less than 1%. The 14 district councils represented high (7), moderate (3), low (1), and very low (3) malaria transmission strata. *Anopheles funestus* s.s. was predominant in high transmission strata in the Lake and South-East zones while *An. arabiensis* was predominant in the low and very low strata in the central corridor though this species was found in most district councils (Fig. 6B). *Anopheles gambiae* s.s. was found low numbers in numbers across most district councils.

### Distribution of man biting rate, sporozoite rates and entomological inoculation rates by species

**Fig. 7:**
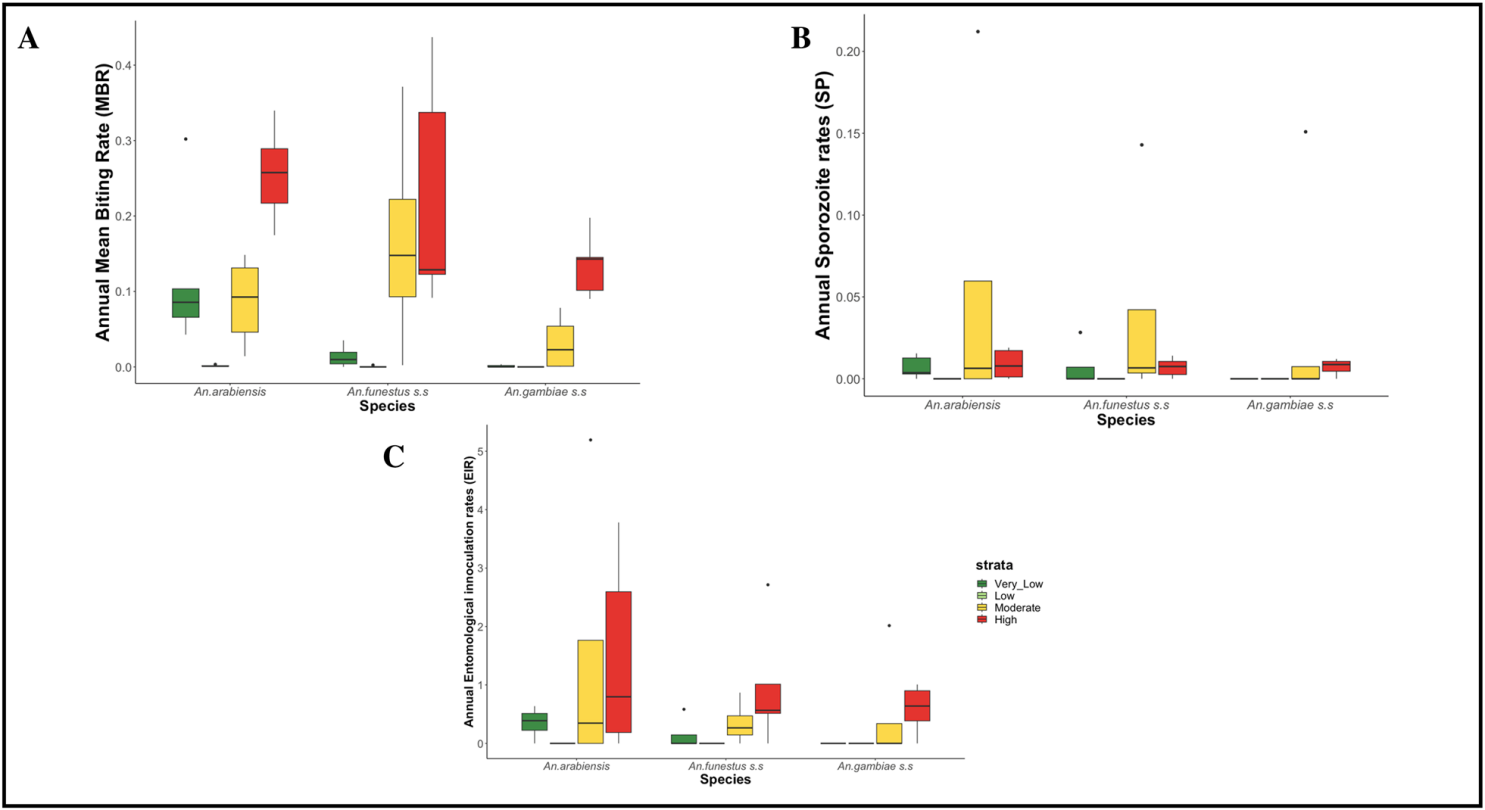
Distribution of Man biting rate (**A**), sporozoite rates (**B**) and entomological inoculation rates (**C**) by Species by epidemiological strata

Table 1 presents a summary of the distribution of sporozoite rates (SR), man biting rates (MBR), and annual entomological inoculation rates (EIR) by malaria vector species. A similar summary is also provided in Fig. 7 based on box plots. The estimated man biting rates (MBR) combined for all the years were 0.07 bites/person/year (95% Confidence Intervals, CI 0.05–0.09) for *An. gambiae* s.s., 0.12 bites/person/year (95% CI 0.09–0.13) for *An. arabiensis*, and 0.12 bites/person/year (95% CI 0.11–0.15) for *An. funestus* s.s.

**Table 1:** Distribution of man biting rate (MBR), sporozoite rates (SR), and annual EIR by malaria vector species

Mosquito species	Year	Total mosquito tested	Pf positive	SR	MBR	Annual EIR
*An. gambiae* s.s.	2017	1023	24	0.023	0.110	1.48
	2018	2320	22	0.009	0.074	0.39
	2019	1061	8	0.008	0.048	0.22
	2020	355	0	0.000	0.035	0.00
	2021	434	2	0.005	0.072	0.21
Average (95% CI)		1038.6	11.2	0.009	0.0678	0.46
*An. arabiensis*	2017	3047	48	0.016	0.164	1.54
	2018	5299	142	0.027	0.128	2.02
	2019	2884	18	0.006	0.098	0.34
	2020	1266	2	0.002	0.094	0.11
	2021	1150	3	0.003	0.095	0.17
Average (95% CI)		2729.2	42.6	0.0108	0.1158	0.836
*An. funestus* s.s.	2017	569	8	0.014	0.123	1.01
	2018	2763	16	0.006	0.089	0.31
	2019	3403	0	0.000	0.154	0.00
	2020	1738	8	0.005	0.129	0.38
	2021	1431	18	0.013	0.158	1.20
Average (95% CI)		1980.8	10	0.0076	0.1306	0.58

*Pf* *Plasmodium falciparum*, *MBR* Man biting rate, *EIR* entomological inoculation rate, *CI* = confidence interval

The estimated sporozoite rates (SR) were 0.009 (95% CI 0.002–0.015) for *An. gambiae* s.s., 0.11 (95% CI 0.093–0.138) for *An. arabiensis*, and 0.115 (95% CI 0.108–0.152) for *An. funestus* s.s. The estimated combined entomological inoculation rates (EIR) were 0.46 infectious bites/person/year (95% CI 0.028–0.928) for *An. gambiae* s.s*.*, 0.836 infectious bites/person/year (95% CI 0.138–1.559) for *An. arabiensis*, and 0.58 infectious bites/person/year (95% CI 0.165–0.971) for *An. funestus* s.s with variations across different malaria transmission strata (Table 1).

The MBR were statistically significant between *An. gambiae* and *An. funestus* (p < 0.05). Higher EIR values for *An. funestus* s.s. compared to *An. arabiensis* were observed in recent years (i.e., 2020 and 2021) based on the data from the selected 14 district councils.

### Distribution of man biting rates, sporozoite rates, and annual entomological inoculation rates by malaria strata

Table 2 presents a summary of man biting rates (MBR), sporozoite rates (SR), and annual entomological inoculation rates (EIR) by malaria strata—a similar summary is also provided in Fig. 7 based on box plots. In high stratum, the MBR was estimated at 0.205 bites/person/year (95% CI 0.156–0.255), SR was 0.008 (95% 0.004–0.013), and EIR was 0.984 infectious bites/person/year (95% CI 0.445–1.526). In very low stratum, the estimated MBR was 0 bites/person/year (95% CI 0–0), SR was 0 (95% 0–0), and EIR was 0 infectious bites/person/year (95% CI 0–0). There were significant differences in MBR between the low and high strata (p = 0.002) and between the moderate and high strata (p = 0.019), Table 2.

**Table 2:** Distribution of man biting rates (MBR), sporozoite rates (SR) and annual EIR by malaria strata

Malaria strata	Year	Total mosquito tested	Pf positive	SR	MBR	Annual EIR
High	2017	3062	46	0.015	0.220	1.93
	2018	5517	72	0.013	0.177	1.35
	2019	2619	18	0.007	0.119	0.49
	2020	2203	3	0.001	0.219	0.13
	2021	2621	17	0.006	0.290	1.02
Average (95% CI)		3204.4	31.2	0.0084 (0.0041–0.0129)	0.205 (0.1559–0.2554)	0.984 (0.4498–1.5261)
Moderate	2017	172	30	0.174	0.019	1.94
	2018	3634	104	0.029	0.117	1.99
	2019	4090	0	0.000	0.000	0.00
	2020	892	7	0.008	0.089	0.42
	2021	149	1	0.007	0.016	0.19
Average (95% CI)		1787.4	28.4	0.0436 (0.0123–0.0989)	0.0482 (0.0071–0.0878)	0.908 (0.1329–1.6676)
Low	2017	4	0	0.000	0.000	0.00
	2018	8	0	0.000	0.000	0.00
	2019	7	0	0.000	0.000	0.00
	2020	13	0	0.000	0.000	0.00
	2021	10	0	0.000	0.000	0.00
Average (95% CI)		8.4	0	0 (0–0)	0 (0–0)	0 (0–0)
Very Low	2017	1401	4	0.003	0.302	0.53
	2018	1223	4	0.003	0.039	0.07
	2019	632	8	0.013	0.029	0.71
	2020	251	0	0.000	0.000	0.00
	2021	235	5	0.021	0.039	0.97
Average (95% CI)		748.4	4.2	0.008 (0.0012–0.0148)	0.0818 (0.0183–0.1802)	0.456 (0.1279–0.7850)

## Discussion

This paper provides a detailed account on the malaria vectors and transmission intensity from five years of malaria vector entomological surveillance in mainland Tanzania. The findings from the analysis indicate that based on morphological identification, out of 63,226 combined mosquitoes reported from 32 district councils, 62% are *An. gambiae* s.l., 33% are *An. funestus*, and 0.05% are other *Anopheles*. Out of 41,383 mosquitoes that were amplified during laboratory analysis based on samples submitted from 14 district councils, 51% are *An. arabiensis,* 33% are *An. funestus* s.s., 15% are *An. gambiae* s.s. Based on 14 qualified district councils, 14,301 out of 29,524 mosquitoes tested, 48% are *An. arabiensis,* 34% are *An. funestus* s.s.,18% are *An. gambiae* s.s.

The estimated man biting rates (MBR) varied across the different mosquito species and strata but on average were significantly higher in *An. gambiae* than *An. funestus* (p < 0.05). The MBR was significantly higher in high stratum than both moderate (p < 0.05) and low (p < 0.01) strata. There were some variations between sporozoite rates (SR) between different species and strata. However, no significant difference between either species or strata can be reported based on the data from the 14 district councils. In addition, the estimated entomological inoculation rates (EIR) were not significantly different across district councils, strata, or species. Several mosquito samples in the very low stratum were positive for sporozoites in 2019 and 2021. This was likely due to an outbreak in district councils in this stratum. Most samples from the very low stratum were negative for sporozoites. This resulted in EIR estimates that were unexpectedly higher in the very low stratum compared to the low stratum district councils. On average, the EIR was < 1 in several district councils in Tanzania which according to Beier et al*.* [28] is an indication that the malaria transmission maybe interrupted in those district councils. The marked decline of EIR estimates might be attributed to deployment of effective malaria control measures including deployment of IRS in the Lake Zone for a couple of years and the scale up of LLINs across the Country.

However, the EIR was > 1 in a number of district councils in moderate and high transmission areas mediated mostly by *An. funestus* s.s. and *An. arabiensis*. The NMCP and partners must maintain and strengthen indoor control interventions targeting *An. funestus* [30], while new tools are needed to address outdoor transmission that is mediated by *An. arabiensis.* Integrated vector management approach should be implemented with interventions such as LSM [31, 32] targeting immature mosquitoes should be considered but with careful planning and deployment based on World Health Organization (WHO) recommendations and/or in country experiences. Also, interventions targeting outdoor biting mosquitoes such as spatial repellents should be considered. Fortunately, LSM is considered a priority intervention in Tanzania and the plans for its implementation are well elaborated in the National Malaria Strategic Plan [33]. In addition, NMCP in collaboration with President’s Office, Regional Administration and Local Government Tanzania (PORALG) and a partner project, Towards Elimination of Malaria in Tanzania (TEMT) is implementing the LSM as a pilot project in Tanga region. The experiences and lessons from the TEMT project and modelling approaches [34, 35] should be considered to guide the scaling up of LSM in Tanzania.

The key findings from MVES are similar to several research-based studies conducted in specific study areas in Tanzania as indicated in selected references [13, 36, 37]. *Anopheles funestus* s.s. and *An. arabiensis* are observed to be more predominant in the Lake and South-East zones respectively, mostly, high transmission stratum. In general, *An. arabiensis* is found in most district councils in higher numbers as compared to *An. funestus *s.s.—with low numbers for *An. gambiae *s.s. across different district councils. *An. funestus *s.s. is becoming a more efficient species with higher EIR values reported in recent years (i.e., 2020 and 2021) as compared to those of *An. arabiensis* also reported in another study [38] in Tanzania. The impact of seasonality is observed across all district councils, in general, the monthly mosquito densities show strong seasonal signals with two peaks after the rainy seasons, although the precise timing of the peaks differs slightly between species and district councils.

During molecular characterization, several mosquito samples were reported as unamplified equivalent to 31.33%. The NMCP in collaboration with in-country research institutions and academia should consider purifying and reanalysing the DNA of ‘unamplified’ samples as a watch for other important vectors including *Anopheles stephensi*. Recently, the WHO issued a vector alert calling for countries in sub-Saharan Africa to increase vigilance for this invasive vector. As Tanzania updates its national vector surveillance framework to integrate *An. stephensi*, as a pre-emptive action against a threat to invasion it will also be important to ascertain that the vector is not already in the country unnoticed. In Sudan, *An. stephensi* was first described in samples that failed in PCR for *An. gambiae *s.l. species identification [39].

The MVES programme is designed to ensure sustainability where community volunteers at the household level are responsible for setting mosquito traps under DVCOs’ supervision. The DVCOs are expected to perform morphological identification, label, and pack the samples, right after the three consecutive days of mosquito collection, ready for the national supervision team to transfer the samples to the laboratory. The national supervision team is expected to visit all district councils on quarterly basis (i.e., 4 times a year), perform supervision, identify, and resolve any field encountered challenges, collect samples, and send them to the laboratory. During the implementation using this approach several challenges were noted including low commitment from some of DVCOs leading to poor reporting of data, misidentification in some mosquito samples by some DVCOs, mismanagement of traps and chargers, and improper sample storage in the field. Also, occasionally the national supervision was conducted three times a year instead of four due to budget constraints leading to delays in sample submission to the laboratory. In addition, there was a fuzzy linkage between field and laboratory data which made tracing back of information to the household or village level not possible.

In addition, methodological limitations in the laboratory analysis are also discussed. Circumsporozoite (CSP) enzyme-linked immunosorbent assays (ELISA) have traditionally been considered the 'gold standard for vector incrimination.' CSP ELISA specifically detects the circumsporozoite protein expressed exclusively by sporozoites, enabling the determination *of P. falciparum* and *Plasmodium vivax* species. However, the CSP ELISA method is known to have limitations. It can detect sporozoites that are still developing in the midgut oocyst of the mosquito abdomen, prior to reaching the salivary glands when the mosquito is considered infectious. Moreover, it has shown high rates of false positives due to cross-reactivity with non-*Plasmodium* antigens, especially in zoophilic vectors where an unidentified heat-labile antigen from animal blood can trigger cross-reactivity. These limitations have underscored the need for more sensitive and robust methods of vector incrimination. Therefore, in this study, the use of a PCR-based method to detect the parasite's mitochondrial (mt) *cox-I* gene was opted, which is preferred over the CSP ELISA method. The PCR-based method has been recognized as more sensitive than the traditional 'gold-standard' CSP ELISA. Although the mt COX-I PCR is not entirely specific for the infectious sporozoite stage, it is still a highly sensitive and robust method for detecting *Plasmodium* DNA in mosquitoes.

Despite these limitation and challenges, the combined dataset from all the district councils, and especially those with consistent data reporting and sample submission to the laboratory, provides an assessment on malaria vector species composition, their abundance and seasonality, place of biting, host preference (vector behavior), and entomological inoculation rates for each species by strata. These entomological indicators are important in assessing the performance of previously deployed vector control interventions over time and in providing guidance on re-deployment going forward.

As a way forward, several adjustments are being made to streamline the MVES and improve data quality with lessons from the five years of experience. As an example, change in data entry template was done to ensure that cells are locked, and the template cannot be modified on the ground—starting from 2021 there is an improvement in data quality. Along a similar vein, the NMCP will need to finalize its plan to deploy an electronic database system to manage both field and laboratory data with proper data linkage to the household level. Given the vast size of the country and heterogeneity in malaria transmission, an electronic database system will facilitate monitoring of data reporting progress, recording data electronically even with no internet connectivity, ensure accountability at different levels, and provide interactive dashboards to visualize data in real time by programme management. The system will also ease the data sharing with DHIS2 and/or other data repositories/platforms in line with the NMCP desire to link entomological with epidemiological data and all information related to malaria control elimination strategies in the country. The generic schema described [40] provides key principles for designing and developing entomological databases that can be used to support diverse entomological studies including routine surveillance conducted by NMCPs. One such electronic system is Mosquito Database Management System (MosquitoDB), www.mosquitodb.io, that may be adapted by NMCPs to manage both field and laboratory data.

It is important to ensure that in addition to having a robust electronic entomological system, DVCOs are constantly trained and are committed to collect and record data timely. A suitable approach and method should be deployed to make sure that the information on mosquito resting behaviours is also recorded [40]. In-line with these recommendations for improvements, NMCP should consider increasing the number of district councils to ensure that it is well positioned to monitor invasive mosquito species including *An. stephensi*.

The MVES system in Tanzania sets a good example to other countries either struggling to maintain or planning to establish malaria vector entomological surveillance systems. The experiences to be shared are particularly on the MVES’s methodology including the criteria provided for selecting sentinel sites.

## Conclusion

This work provides an update on malaria vectors in Tanzania from 2017 to 2021 based on different transmission strata. *An. arabiensis* is still the most abundant vector species found across most district councils, but *An. funestus *s.s. is equally contributing to malaria transmission especially in high transmission stratum. The NMCP and partners must maintain and strengthen indoor control interventions targeting *An. funestus *s.s. and *An. gambiae *s.s., but equally important to consider targeting outdoor transmission that is mediated by *An. arabiensis.* The intervention such larval source management (LSM) targeting immature mosquitoes and interventions targeting outdoor biting mosquitoes should be considered but with careful planning and deployment. In addition, NMCP should adopt recommendations provided to ensure proper implementation of the MVES program from the ground while ensuring management of quality entomological data. The challenges and lessons highlighted from MVES Tanzania may be used to guide other countries with plans to establish their own MVES programme.

## Data Availability

The dataset is available upon a request made to the NMCP in Tanzania.
